# Integrating a Machine Learning System Into Clinical Workflows: Qualitative Study

**DOI:** 10.2196/22421

**Published:** 2020-11-19

**Authors:** Sahil Sandhu, Anthony L Lin, Nathan Brajer, Jessica Sperling, William Ratliff, Armando D Bedoya, Suresh Balu, Cara O'Brien, Mark P Sendak

**Affiliations:** 1 Trinity College of Arts & Sciences Duke University Durham, NC United States; 2 Duke University School of Medicine Durham, NC United States; 3 Social Science Research Institute Duke University Durham, NC United States; 4 Duke Institute for Health Innovation Durham, NC United States; 5 Division of Pulmonary, Allergy, and Critical Care Medicine Duke University School of Medicine Durham, NC United States; 6 Department of Medicine Duke University School of Medicine Durham, NC United States

**Keywords:** machine learning, sepsis, qualitative research, hospital rapid response team, emergency medicine

## Abstract

**Background:**

Machine learning models have the potential to improve diagnostic accuracy and management of acute conditions. Despite growing efforts to evaluate and validate such models, little is known about how to best translate and implement these products as part of routine clinical care.

**Objective:**

This study aims to explore the factors influencing the integration of a machine learning sepsis early warning system (*Sepsis Watch*) into clinical workflows.

**Methods:**

We conducted semistructured interviews with 15 frontline emergency department physicians and rapid response team nurses who participated in the Sepsis Watch quality improvement initiative. Interviews were audio recorded and transcribed. We used a modified grounded theory approach to identify key themes and analyze qualitative data.

**Results:**

A total of 3 dominant themes emerged: perceived utility and trust, implementation of Sepsis Watch processes, and workforce considerations. Participants described their unfamiliarity with machine learning models. As a result, clinician trust was influenced by the perceived accuracy and utility of the model from personal program experience. Implementation of Sepsis Watch was facilitated by the easy-to-use tablet application and communication strategies that were developed by nurses to share model outputs with physicians. Barriers included the flow of information among clinicians and gaps in knowledge about the model itself and broader workflow processes.

**Conclusions:**

This study generated insights into how frontline clinicians perceived machine learning models and the barriers to integrating them into clinical workflows. These findings can inform future efforts to implement machine learning interventions in real-world settings and maximize the adoption of these interventions.

## Introduction

Advances in predictive analytics and machine learning offer an opportunity to improve the diagnosis and management of acute conditions. A prominent use case for machine learning in health care is sepsis, a leading cause of death in US hospitals [1], which accounts for 1.7 million hospitalizations [2] and costs the US health system US $23 billion annually [3]. Machine learning algorithms have been shown to outperform traditional screening scores in early sepsis detection [4]. Despite the rise in competing models and products for early sepsis detection [5], few machine learning models have been implemented as part of clinical care [6-8]. As a result, little evidence exists on the optimal integration of these sepsis models and other machine learning models into clinical workflows [9].

Early detection and treatment of sepsis is essential to decrease patient mortality [10]. Implementing standardized bundles can help ensure timely and proper sepsis care and has been associated with reductions in mortality [11-13]. Despite consensus that sepsis treatment bundles improve patient outcomes, only 49% of patients in US hospitals receive appropriate care [14]. The reasons for low compliance include the lack of a gold standard for sepsis diagnosis and the difficulty of rapidly mobilizing resources needed to treat individuals suspected of having sepsis [15,16].

Clinical decision support (CDS) systems may play a role in improving bundle compliance and delivery of timely treatment. CDS sepsis early warning systems leverage electronic health information to continuously stratify patients for the risk of sepsis and alert clinicians [17-22]. Unfortunately, many sepsis CDS systems fail to improve outcomes because of poor diagnostic accuracy and program implementation [23-25]. Eliciting perspectives directly from clinicians using such models can identify real-world barriers and facilitators impacting implementation efforts. Despite a growing body of literature on both physician perspectives of sepsis CDS implementation [26-28] and sepsis machine learning algorithms, more research is needed to understand clinician views on *black box* machine learning models that do not explain their predictions in a way that humans can understand [29,30]. To address this gap in the literature, this study identifies factors that affect the integration of a *black box* sepsis machine learning system into the workflows of frontline clinicians.

## Methods

### Setting

This study analyzes the implementation of the Sepsis Watch program at the Duke University Hospital (DUH). DUH is the flagship hospital of a multi-hospital academic health system with approximately 80,000 emergency department (ED) visits annually. According to our institutional definition for sepsis, over 20% of adults admitted through the DUH ED develop sepsis [31], and nearly 68% of sepsis occurs within the first 24 hours of hospital encounter [32].

### Program Description

In a previous study, we designed a digital phenotype for sepsis using clinical data available in real time during the patient’s hospital encounter. We then developed a deep learning model to predict a patient’s likelihood of meeting the sepsis phenotype within the subsequent 4 hours [33,34]. The model analyzed 42,000 inpatient encounters and 32 million data points. Model inputs included static features (eg, patient demographics, encounter information, and prehospital comorbidities) and dynamic features (eg, laboratory values, vital signs, and medication administrations). The model pulls data from the electronic health record (EHR) and is updated every hour to ensure real-time analysis of sepsis risk.

**Figure 1:**
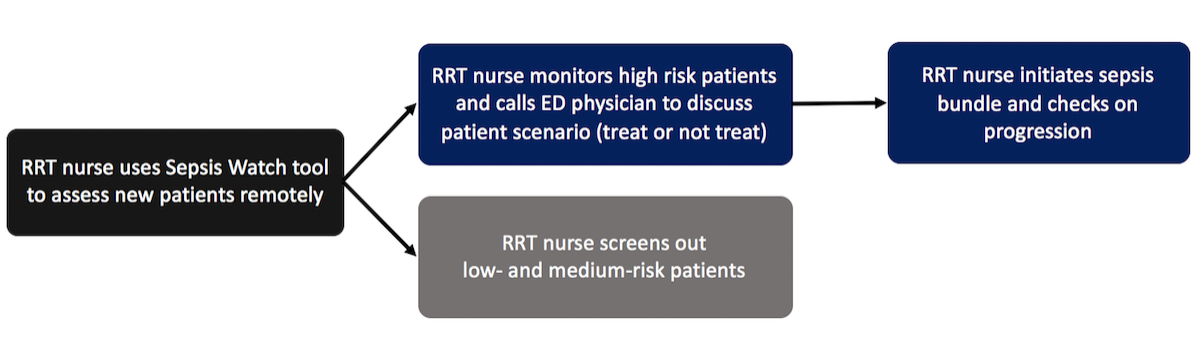
Sepsis Watch workflow. ED: emergency department; RRT: rapid response team.

Concurrent with model development, an interdisciplinary team of clinicians, administrators, and data scientists designed a workflow to translate outputs from the model into clinical action (Figure 1). The team created a web application to display all patients presenting to the ED and their risk of sepsis. In the application, every patient was classified and presented by the model as meeting sepsis criteria (black card), high risk of sepsis (red card), medium risk (orange card), or low risk (yellow card). Rapid response team (RRT) nurses are the primary users of the Sepsis Watch application and remotely monitor all patients in the ED. For patients meeting sepsis criteria or at high risk of sepsis, an RRT nurse conducts a chart review and calls the ED attending physician to discuss the patients’ care pathway. If the attending physician agrees that the patient is likely to have sepsis, the RRT nurse supports the patient care team to ensure that sepsis care bundle items are ordered and completed. After the call, the RRT nurse continues to monitor the completion of the bundle items and follows up as needed with the ED attending physician or ED nurse.

Before implementation, RRT nurses were extensively trained in person in the program workflow and application. ED physicians were informed about the program in faculty meetings and via email. Both nurses and physicians were educated on the model’s aggregate performance measures relative to other methods, and visualizations of individual patient cases were presented to demonstrate how the model could detect sepsis hours before the clinical diagnosis [30]. A full description of the planning and implementation process can be found elsewhere [32].

### Study Design

A team of clinicians and social science researchers cocreated 2 interview guides: one for ED attending physicians and one for RRT nurses. The guide was designed to walk participants through each step of the workflow and probe for the associated barriers and facilitators. Subsequent questions in the interview guides covered training and dissemination of the new program, areas for improvement, and perceived utility. The guides were informed by the situational awareness model, which differentiates among 3 levels of situational awareness: (1) perception of relevant information, (2) comprehension of that information, and (3) anticipation of future events based on that information [35]. Although we drew from the situational awareness model to help support a high-level structure, we did not aim to examine the effect of Sepsis Watch on these levels or underlying mechanisms given our inductive and exploratory study approach. The interview guides were piloted among 3 clinicians to inform improvements to specific questions and overall structure.

ED leaders and RRT leaders invited physicians and RRT nurses to participate in semistructured interviews. As an operational project, participation was fully voluntary. From January 2019 to April 2019, we recruited 7 ED physicians (n=7; Table 1) and 8 RRT nurses (n=8; Table 2) to participate in the semistructured interviews. Although we used a convenience sampling approach to recruit participants, Tables 1 and 2 demonstrate how individual participants represent a diverse sample with regard to demographics, experience, and involvement in the design of the program. Of the 15 participants, 4 were involved in the design and development of Sepsis Watch.

**Table 1:** Characteristics of rapid response team nurse participants.

Participant	Experience as an RRT^a^ nurse	Experience as a nurse	Was the participant involved in program development?
RRT nurse 1	7 months	4 years	No
RRT nurse 2	4 years	13 years	Yes
RRT nurse 3	5 years	10 years	No
RRT nurse 4	4 years	10 years	No
RRT nurse 5	3 years	5 years	No
RRT nurse 6	5 years	30 years	Yes
RRT nurse 7	3 years	4 years	Yes
RRT nurse 8	4 years	10 years	No

^a^RRT: rapid response team.

**Table 2:** Characteristics of emergency department attending participants.

Participant	Experience as attending physician at pilot site	Experience as attending physician	Was the participant involved in program development?
ED^a^ attending 1	5 years	5 years	No
ED attending 2	2 years	2 years	No
ED attending 3	13 years	13 years	No
ED attending 4	5 years	5 years	Yes
ED attending 5	8 years	16 years	No
ED attending 6	9 months	9 months	No
ED attending 7	6 years	3 years	No

^a^ED: emergency department.

All interviews were conducted in person and face to face by the first author. Data collection started 4 months into the implementation of Sepsis Watch to give participants enough time to reflect on the initial rollout and describe any changes in workflow and perceptions. Following a semistructured interview format, participants were asked the same questions delineated in the interview guide, with flexibility for follow-up questions and probes. The interviews averaged 35 min in duration. Interviews were recorded with the written consent of the participants and transcribed verbatim. In the qualitative analytic process, data collection was terminated when no new themes or insights emerged (ie, thematic saturation) across both participant groups [36].

To analyze the transcripts, we followed a modified grounded theory approach, a widely used and established analytic method in social science research [37]. Grounded theory provides a systematic approach to derive and classify themes from qualitative data, such as interview transcripts. This approach employs a coding process to organize qualitative data, in which text is labeled with *codes* or short phrases that reflect the meaning of sentences or paragraphs [38]. These codes are then used to generate higher-level themes that emerge as the major study findings.

In our study, coding was conducted in 3 phases. In the first phase, we used line-by-line coding to create tentative open codes closely grounded in the raw data. In the second phase, focused coding was employed to create higher-level categories and subcategories from the open codes. In the third phase, we selectively defined relationships among various categories. The final codebook was discussed and reviewed with members of the research team. Data were analyzed using NVivo Qualitative Data Analysis Software (version 12, QSR International). We queried codes to identify the most prevalent themes among the 2 participant groups. This study was approved by the Duke University Health System Institutional Review Board (Protocol ID: Pro00093721).

## Results

A variety of themes emerged as important factors that shaped the integration of Sepsis Watch into routine clinical care (Table 3). Factors were grouped into 3 thematic areas: (1) perception of utility and trust, (2) implementation of Sepsis Watch processes, and (3) workforce considerations. For each area, we describe the corresponding subthemes with representative quotations.

**Table 3:** Thematic area and corresponding subthemes.

Thematic area	Subtheme
Perception of utility and trust	Trust and accuracy Perception of machine learning Context-specific utility
Implementation of the Sepsis Watch program	Tool layout and design Value of human communication Nurse strategies Information flow challenges Gaps in knowledge and understanding
Workforce considerations	A new role—Sepsis Watch nurse Skills and capabilities required for success

### Perception of Utility and Trust

This area focuses on themes related to clinicians’ attitudes toward the Sepsis Watch program, including trust and accuracy, broader perceptions about the role of machine learning in clinical practice, and the settings in which the program was perceived to be the most useful. Representative quotations are presented in Table 4.

**Table 4:** Representative quotations on perceived utility and trust.

Subtheme	Quote
Trust and accuracy	“Sepsis Watch is very good at predicting patients and identifying patients who are septic and...we’ve had a lot of patients here that have actually come to our [CICU] unit from the ED^a^ who have popped up on Sepsis Watch.” [RRT^b^ nurse] “Blood cultures seem to weigh very heavily in the algorithm...I can pretty much bet you money that every single time I order blood cultures on somebody, sixty minutes later I’ll get a phone call from Sepsis Watch that says they tripped positive...it means I was thinking about infection but I wasn’t worried enough to pursue the true sepsis bundle.” [ED physician] “I had at least two patients who went to the ICU that I never got a Sepsis Watch call for, at all. So, I don’t know how those got missed...The rest of them, so a lot of the false positives were like...COPD exacerbation or something like that.” [ED physician] “The initiative...just creates a lot more vigilance...I almost feel like I’m very cognizant of sepsis and almost like, imagining the Sepsis Watch people upstairs like, looking down on me...I’m honestly like, just waiting for their call. Like, can you imagine like, I was like, oh this must be them. So, in some ways I think that’s good, that it has fostered vigilance.” [ED physician]
Perception of machine learning	“I think a big part of people not understanding [Sepsis Watch], including actually the ED doc, is if vitals are stable. We’re not gonna treat because they look stable. I know but we’re trying to catch it before it’s unstable. And that’s the biggest piece people don’t get...fact that it’s predictive like, hammering that in will help people see…we’re trying to prevent the decline.” [RRT nurse] “Most people don’t know much about [machine learning] and there’s always this idea of like, you can’t replace me and my training and that I’m standing in front of the patient telling you if they’re septic or not.” [ED physician]
Context-specific utility	“It’s probably a way more useful tool, not in the ED. In the ED, all we think about all the time is sepsis cause it’s such a big part of our practice. So, that’s why I think it doesn’t apply well to us, but it would apply well in other settings where they don’t think about or see or miss the bundle more often.” [ED physician]

^a^ED: emergency department.

^b^RRT: rapid response team.

#### Trust and Accuracy

RRT nurses, the primary users of the Sepsis Watch tool, spoke positively about the accuracy of the Sepsis Watch model to detect sepsis. When asked how the Sepsis Watch tool faired against other CDS tools such as the National Early Warning Score that they had previously used [23], the RRT nurses overwhelmingly described the relative advantage of Sepsis Watch, both in diagnostic accuracy and data visualization. However, ED physicians described relatively less trust in the model. They thought certain components of the Sepsis Watch algorithm were too heavily weighted (eg*, blood cultures*). Physicians also noted that Sepsis Watch both missed some important sepsis cases and had false positives. To build more trust in the model, several physicians requested feedback on the success of Sepsis Watch and the specific cases that they missed but were detected by Sepsis Watch.

RRT nurses generally believed that the Sepsis Watch program improved sepsis detection and bundle compliance, especially because the implementation team provided them with data on the success of the program. ED physicians reported that Sepsis Watch had increased the vigilance and proactive culture about sepsis in the ED but were overall less positive than the RRT nurses. ED physicians described the difficulty in achieving the perfectly timed intervention in which the ED attending physician has had time to evaluate the patient but has still not diagnosed sepsis or completed the bundle items.

### Perception of Machine Learning

Both RRT nurses and ED physicians said that they lacked the knowledge and understanding required to assess the validity of the machine learning model. Nurses reported feeling uncomfortable reviewing and assessing high-risk patients with minimal information and would often wait for more information to populate the medical record before having the confidence to call the ED physicians. Physicians also lacked knowledge about the model and the predictive nature of the model.

When asked about the role of machine learning in health care more broadly, physicians had varied responses. Some physicians noted a lack of knowledge, fear of overstepping, and resistance to change in medicine as potential barriers. Other physicians saw an opportunity to introduce machine learning to operations and logistics problems before CDS.

#### Context-Specific Utility

ED physicians felt that they were not necessarily the appropriate target adopters for Sepsis Watch. Respondents felt that ED attending physicians at large academic health centers were particularly adept at identifying and treating sepsis. Instead, they perceived Sepsis Watch to be most useful for residents who were still developing clinical skills, low-resource community settings, or hospitals with a poor track record for treating sepsis.

### Implementation of Sepsis Watch Processes

This thematic area focuses on the implementation of the Sepsis Watch program, including the use of the tool and the interactions between RRT nurses and ED physicians. Although the nurses created their own communication strategies to facilitate interaction, barriers to positive interactions included challenges in information flow and gaps in understanding and knowledge. Representative quotations are presented in Table 5.

**Table 5:** Representative quotations on the implementation of Sepsis Watch processes.

Subtheme	Quote
Layout and design	“It’s just easy to navigate. You start at triage and go through the different tabs. The colors are easy...You quickly glance at it and you already have an idea of what you’re getting yourself into...If you’re used to navigating an iPhone, it’s pretty easy to just figure it out.” [RRT^a^ nurse] “I start to really go through the patient’s chart and see what they presented to the emergency room for you know, what was their complaint, what’s their past history, and then I’ll look at their lab values and things like that and vitals and medications and stuff...The biggest thing I look for is the notes you know that the ED^b^ staff are writing. You know, that kind of guides a lot.” [RRT nurse]
Value of human communication	“No matter how good the technology is, if the interface is bad no one’s going to use it and then they’re going to interpret that as the technology is bad...we use the RRT...like an air traffic controller in an airport that gets all this stuff, consolidates it and calls it out to the right people, until we figure out a way to do it through the computer interface.” [ED physician]
Nurse strategies	“This is how it goes. ‘Hey, this is [person’s name] from Sepsis Watch. How are you? Good. Okay, I’m calling about Mr Wallace in A-15. He’s popped up at high risk for sepsis. I see that you know, he came in complaining of a cough. I see that you’ve already done like, a lactate, antibiotics. Are you thinking sepsis?’ I try to put a piece of information to show I’ve done a chart review to show that this is not like, a cold call, that I’ve actually looked.” [RRT nurse]
Informational flow challenges	“If [the ED physicians] are busy with other patients, sometimes you cannot get communication with them on the first point of contact, so on your first phone call, they may be running a code in the resuscitation bay...then you have to wait about like, an hour or two to kind of get in touch with them.” [RRT nurse] “It’s an interruption. I mean it’s a random call at a random time that’s completely disruptive to workflow. Every single call we get is completely disruptive to workflow. And when it’s not giving me any new information, it’s even less helpful.” [ED physician] “It would be hard to escalate to the ED physicians because we don’t work with them, we’re not there, we don’t have that relationship with them. They don’t know who we are, they don’t really know what we do, so I think for me, then to be saying I feel like you need to start this patient on antibiotics...that wouldn’t go down too well...If you were like, down, physically down in the ED with them, I think that would be a different case scenario.” [RRT nurse] “Part of the problem is, ED is such a team-based approach that it’s often that you’re so busy that I’m sort of doing my round around the ED caring for people and the resident’s doing theirs, and the nurses doing theirs that you might not overlap frequently enough or adequately enough to convey that information to the people that need to know. For me to have to track them both down to give them that information would be burdensome and that’s what would get in the way of flow in the ED.” [ED physician]
Gaps in knowledge and understanding	“In the beginning it was very difficult making those phone calls because I don’t think that they understood exactly what Sepsis Watch was. There was a lot of like, ‘who are you, what are you doing, is this is lawsuit type of thing?’ They were worried...that if they decided not to treat...and then it turned into sepsis, that they were worried about potentially getting sued for malpractice.” [RRT nurse] “At first there was a little bit of unwieldiness with the actual bucket that we could sort the patients into, so in other words, what does it mean to place them into the [sepsis bundle] protocol, continue to watch them, or to say no, the source is not septic, they don’t need to be watched any longer. But I think as time’s gone on now, we’re more comfortable with the different answers that they’re looking for and that Sepsis Watch nurses are more comfortable guiding us to an answer.” [ED physician] “I think there’s some areas for [ED physicians] to learn because a few questions I will get are like ‘why does [Sepsis Watch] say they’re high-risk because they don’t look septic here.’ Obviously, I don’t know exactly why the app is populating them that way, so I think if they understood that we don’t have all the bits of information that are making them a red card or a black card or yellow or orange...I just have how the computer model populates them into which color and I’m kind of going from there.” [RRT nurse]

^a^RRT: rapid response team.

^b^ED: emergency department.

#### Layout and Design

RRT nurses frequently complimented the Sepsis Watch application for being easy to use and well designed. They described the benefits of visually delineating sepsis risk into colors (eg, red cards as high risk, orange cards as medium risk) and tracking patients across distinct tabs (eg, patients to be triaged, screened out for sepsis, and those in the sepsis bundle). Although RRT nurses use the Sepsis Watch dashboard to monitor important sepsis signs and symptoms (eg, lactate and white blood count), the EHR remains a valuable source for additional information. Some RRT nurses felt more comfortable with the patient’s chart and reported aggregating information presented in both systems.

#### Value of Human Communication

Both ED physicians and RRT nurses described the benefits of having RRT nurses as the effector arm of Sepsis Watch, especially in comparison with the more traditional *best practice alerts* (BPAs) through the EHR. Physicians described how BPAs frequently slow them down and that they are more likely to ignore the BPAs. In contrast, physicians reported that workflow interruptions from human interaction cause less alarm fatigue and get their attention immediately.

#### Nurse Strategies

To facilitate conversations with physicians, RRT nurses developed their own communication and workflow strategies. For example, rather than calling the ED physician for each patient with sepsis or for a high-risk case, nurses often grouped multiple patients together by area of the ED to minimize the number of calls. Similarly, RRT nurses avoided calling ED physicians before shift changes.

During the phone call itself, some RRT nurses ask “how are you” or “is this a good time to call” to gauge the physician’s busyness before they continue the conversation. Other nurses presented information from their chart review to demonstrate that they had a working knowledge of the patient’s case. Many RRT nurses cited the importance of being succinct, direct, and polite to maximize the chances of a positive interaction.

#### Informational Flow Challenges

Although most RRT nurses did not face many barriers in reaching the ED physicians on the phone, some described challenges. Nurses often described how the busy workflows of the ED physicians, coupled with the remote monitoring nature of the RRT, could impede information flow. For example, 1 nurse described challenges in calling physicians amid resuscitation efforts. Nurses hypothesized that communicating with ED physicians might be easier in person than via phone. Furthermore, nurses reported that a lack of working relationships between the ED physicians and RRT nurses before Sepsis Watch made it challenging to build rapport and communicate freely.

Physician respondents noted that calls, although brief, were still interruptions in their busy workflows, which decreased their receptivity for calls from the RRT nurse. In addition, physicians felt that targeting calls about sepsis detection and treatment to the ED attending physicians only required them to disseminate the information to the entire ED care team, such as residents and nurses.

#### Gaps in Knowledge and Understanding

RRT physicians reported that at the start of the program, the ED physicians were unfamiliar with the purpose of the program, the role of the RRT nurse, and the flow of the call. This initial unfamiliarity might have resulted in confusion and misunderstanding. For example, RRT nurses heard from some physicians that they feared being increasingly liable and accountable for proper sepsis treatment with Sepsis Watch rollout. Over time, collaboration between RRT nurses and ED physicians improved.

Even though the Sepsis Watch tool does not inherently provide explanations for risk scores, RRT nurses were sometimes asked to explain the risk score. This created a mismatch between what ED physicians and RRT nurses understood about the technology.

### Workforce Considerations

This thematic area describes the workforce implications of the Sepsis Watch program. The program required the creation of a new professional *Sepsis Watch Nurse* role to translate the machine learning algorithm to the patient’s bedside. It is also important to identify the skills and capabilities needed for nurses to successfully perform the duties of the new role. Representative quotations are presented in Table 6.

**Table 6:** Representative quotations on workforce implications.

Subtheme	Quote
A new role — Sepsis Watch nurse	“It’s been enlightening. You are Sepsis Watch nurse. You are watching sepsis you know, in the ED^a^ and it’s cool you know, it’s a totally new job title under the RRT^b^ role and a new responsibility and one I welcome. I think it’s really good and I think having a nurse with good clinical judgement, hopefully, as being that second check.” [RRT nurse] “I would rather be looking at this than be walking around the unit doing turns, pulling up, boosting, cleaning, and putting out fires on the unit. So, this workflow has been nice like, it allows me to step back and use my mind in a different way.” [RRT nurse] “We’re not here to contradict what they’re already doing. If they tell me that they’re not worried about sepsis, I don’t disagree with them...I don’t try to argue with them. They are the physician. They’re the ones that know the patient. I’m looking at a computer screen. I don’t actually see the patients themselves.” [RRT nurse]
Skills and capabilities required for success	“I think if you have a good clinical background and are familiar with sepsis and you’re kind of familiar with how to treat sepsis and stuff that you can probably perform sepsis watch. I don’t know that you necessarily have to be an RRT nurse...Sepsis Watch is so specific, if you’ve got a good gen[eral] med[icine] background, I think you could probably serve as a good sepsis watch nurse.” [RRT nurse] “I think getting people who only want to do it would be helpful. I think you’ll find enough people who would want to do it I think making it not mandatory for people who don’t want to do it. Recruit some people who do. Management support and buy in and hey, this is your job, it’s important. And positive feedback as far as results, statistics.” [RRT nurse]

^a^ED: emergency department.

^b^RRT: rapid response team.

#### A New Role: Sepsis Watch Nurse

RRT nurses took pride in their new role of a *Sepsis Watch Nurse*, especially given their participation in the program design and pilot implementation. More specifically, RRT nurses enjoyed the investigative and diagnostic role of the Sepsis Watch role. Although RRT nurses are empowered with information through Sepsis Watch, they recognized the boundaries of their own scope of practice and the need to continue to respect the professional autonomy of physicians.

#### Skills and Capabilities Required for Success

When asked about the skills and knowledge needed to be a good Sepsis Watch nurse, the RRT nurses mentioned good clinical judgment, knowledge of sepsis, and critical care experience. If nurses are unfamiliar with sepsis, they might rely too heavily on the model without using their own critical thinking skills. RRT nurses also explained the importance of strong communication skills to confidently speak with attending physicians whom they may not personally know. Although the Sepsis Watch nurse has to interact with a web-based dashboard, RRT nurses thought that strong computer skills were not necessary for the role, given the simplicity of the app. RRT nurses also recommended recruiting nurses interested in the role and the need to create buy-in through continuous feedback.

## Discussion

In our study, we conducted interviews with ED physicians and RRT nurses to understand the factors affecting the integration of a machine learning tool into clinical workflows. We found 3 main thematic areas: (1) perception of utility and trust, (2) implementation of Sepsis Watch processes, and (3) workforce considerations, with 10 corresponding subthemes. Taken together, our findings show how RRT nurses can effectively monitor the outputs of a machine learning model and communicate their assessment to ED physicians. To our knowledge, this is the first qualitative research study to investigate the real-world implementation of a machine learning sepsis early warning system in practice.

RRT nurses had positive impressions of the layout and design of the Sepsis Watch tool. This may be partially explained by the participatory approach with which the Sepsis Watch solution was built. Clinicians provided frequent input from the design of the tool to its broader use in clinical workflows. Clinician preferences were incorporated to optimize the ease of use and utility for end users [39]. For example, the visual display of the risk of sepsis was simplified from a continuous risk scalar value into 3 brightly colored categories of risk (low, medium, and high) to reduce cognitive burden [26]. Furthermore, the simplicity of the tool allowed RRT nurses to integrate Sepsis Watch into their current clinical workflow instead of replacing workflows. The RRT nurses described how they still used the EHR and their own clinical judgment skills to contextualize the model outputs.

We also found that both RRT nurses and ED physicians had very limited prior exposure to machine learning–based CDS systems. The lack of machine learning foundational knowledge and firsthand experience made it more difficult for clinicians to trust the Sepsis Watch algorithm. For example, clinicians often felt uncomfortable trusting the Sepsis Watch prediction when they could not *see* clear signs and symptoms in their patients. Some physicians reported the need to know why and how the model predicted the outcome. Similarly, 2 previous studies examining predictive alerts for sepsis suggested that the perceived utility decreased when the model frequently identified clinically stable patients [27,28]. They also found that false positives from other nonsepsis etiologies could increase alarm fatigue. Thus, product developers must consider how limited model explainability and false positives threaten clinicians’ trust in model outputs, particularly for patients without visible clinical symptoms. At the same time, the goal should be to optimize rather than maximize trust, in which clinicians maintain some skepticism of a tool’s capabilities to prevent overreliance [40]. For example, we learned that even when physicians did not trust a model output, they still reported paying closer attention to a patient’s clinical progression over time or ordering tests more quickly.

Despite these challenges, we also found that positive experiences with the tool and human connections improved clinician acceptance. RRT nurses described that their trust in the model increased from their personal experiences as the algorithm successfully predicted patients with sepsis. Physicians suggested that receiving feedback on patients with sepsis who they had personally missed diagnosing but who were correctly identified by Sepsis Watch would build trust in the model. Future implementation efforts may incorporate feedback loops to improve clinician adoption of machine learning models. As machine learning products become more widespread, health professional schools should incorporate foundational machine learning courses into their curriculum to build baseline literacy [41]. Health care organizations should provide training and educational resources and conferences for their existing clinical staff [42]. Model developers should develop clear product labels to help clinicians understand when and how to appropriately incorporate machine learning model outputs into clinical decisions [43].

As health systems start to implement *black box* models as part of routine care, this study shows the feasibility of leveraging a small team of nurses to communicate machine learning outputs to a larger cohort of ED physicians. Previous attempts at sending automated CDS alerts directly to the treating provider have been associated with high levels of alert fatigue [23]. In our program, RRT nurses mitigate alarm fatigue by screening the patients first, holding the providers more accountable for meeting bundle requirements, and by adding a human connection to the model output. Adding a human intermediary has its own challenges, such as interrupting busy ED workflows and additional delays with remote monitoring. For example, despite the model’s high predictive value within the first hour of ED presentation [44], nurses sometimes waited for more clinical information to populate in the medical record before calling physicians about patients flagged as being at high-risk for sepsis. In future iterations of the program, these human-made delays need to be anticipated and addressed through training or workflow design to ensure patient safety.

Future programs that deploy clinicians as an *effector arm* for model outputs must also consider how to best recruit, train, and deploy their machine learning–ready workforce. We found that creating a new, specialized role for the program allows nurses enough time and patient volume to build unique expertise and effective strategies to communicate with physicians. Recruiting clinicians with strong interest in the program, familiarity with sepsis, and strong communication skills is critical.

We were able to use the findings from these interviews to drive program improvements and to inform the scale. For example, to improve the user interface of the tool, we allowed for more space for free text comments from nurses, and we de-emphasized visual displays of model *trend lines* for sepsis risk. To streamline the workflow and reduce the burden on ED physicians, we no longer required nurses to call ED physicians if they had already clearly ruled out sepsis or started treatment. We also identified broader strategies to build trust and accountability in machine learning tools, such as educating clinicians on model performance and utility within the local context, respecting professional discretion, and engaging end users early and often throughout program design and implementation [30].

### Limitations

This study has several limitations. First, its findings need to be generalized with caution to other settings or applications that differ in organizational structure, capacity, and professional norms and practices. Other settings that also use a participatory approach to intervention development may result in unique tool layouts or workflows. Second, the study largely focuses on workflow integration and does not explore the multi-year planning and stakeholder engagement process crucial for the successful launch of Sepsis Watch. Similarly, only frontline clinicians were interviewed despite the large number of stakeholders involved in project development and maintenance (eg, organizational leadership, hospital administrators, data scientists) or impacted directly by the program (ie, patients; [45,46]). However, for frontline clinicians, having 2 distinct respondent groups in the study allowed for triangulation and strengthening of the analysis. Finally, although we included clinicians involved in the development of the Sepsis Watch program in our sample, given their unique expertise and insight, their participation could have biased the findings to frame Sepsis Watch more positively. Thus, further studies with a larger sample size may use survey approaches to quantify factors influencing the adoption of machine learning CDS tools and examine variations by clinician characteristics, including involvement in program development. Such approaches should consider using well-studied theoretical models for instrument development, such as the Unified Theory of Acceptance and Use of Technology [47].

### Conclusions

Although previous studies have studied factors affecting the implementation of CDS tools [48], the use of black box models in health care settings presents unique challenges and opportunities related to trust and transparency. Previous studies exploring the implementation of these models have focused on surveying clinician perspectives on *future* rollout [42,49-51]. Unfortunately, these studies do not uncover providers’ real-life experiences using artificial intelligence tools in current practice. More research is needed to understand the real-world barriers and facilitators to the design and implementation of machine learning products. Understanding how these factors interact in diverse contexts can inform implementation strategies to ensure adoption. Although we used our findings to inform program improvements locally, our learning can help other health organizations that are planning to integrate machine learning tools into routine practice.

## Abbreviations

BPA: best practice alert
CDS: clinical decision support
DUH: Duke University Hospital
ED: emergency department
EHR: electronic health record
RRT: rapid response team

## References

[1] Rhee C, Jones TM, Hamad Y, Pande A, Varon J, O'Brien C, Anderson DJ, Warren DK, Dantes RB, Epstein L, Klompas M, Centers for Disease Control and Prevention (CDC) Prevention Epicenters Program (2019). Prevalence, underlying causes, and preventability of sepsis-associated mortality in US acute care hospitals. JAMA Netw Open.

[2] Rhee C, Dantes R, Epstein L, Murphy DJ, Seymour CW, Iwashyna TJ, Kadri SS, Angus DC, Danner RL, Fiore AE, Jernigan JA, Martin GS, Septimus E, Warren DK, Karcz A, Chan C, Menchaca JT, Wang R, Gruber S, Klompas M, CDC Prevention Epicenter Program (2017). Incidence and trends of sepsis in US hospitals using clinical vs claims data, 2009-2014. J Am Med Assoc.

[3] Torio CM, Moore BJ National Inpatient Hospital Costs: The Most Expensive Conditions by Payer, 2013: Statistical Brief #204. Healthcare Cost and Utilization Project (HCUP).

[4] Islam MM, Nasrin T, Walther BA, Wu C, Yang H, Li Y (2019). Prediction of sepsis patients using machine learning approach: a meta-analysis. Comput Methods Programs Biomed.

[5] Fleuren LM, Klausch TL, Zwager CL, Schoonmade LJ, Guo T, Roggeveen LF, Swart EL, Girbes AR, Thoral P, Ercole A, Hoogendoorn M, Elbers PW (2020). Machine learning for the prediction of sepsis: a systematic review and meta-analysis of diagnostic test accuracy. Intensive Care Med.

[6] Giannini HM, Ginestra JC, Chivers C, Draugelis M, Hanish A, Schweickert WD, Fuchs BD, Meadows L, Lynch M, Donnelly PJ, Pavan K, Fishman NO, Hanson CW, Umscheid CA (2019). A machine learning algorithm to predict severe sepsis and septic shock: development, implementation, and impact on clinical practice. Crit Care Med.

[7] McCoy A, Das R (2017). Reducing patient mortality, length of stay and readmissions through machine learning-based sepsis prediction in the emergency department, intensive care unit and hospital floor units. BMJ Open Qual.

[8] Shimabukuro DW, Barton CW, Feldman MD, Mataraso SJ, Das R (2017). Effect of a machine learning-based severe sepsis prediction algorithm on patient survival and hospital length of stay: a randomised clinical trial. BMJ Open Respir Res.

[9] Sendak D, Arcy J, Kashyap S, Gao M, Corey K, Ratliff B, Balu S (2020). A path for translation of machine learning products into healthcare delivery. EMJ Innov.

[10] Nguyen HB, Corbett SW, Steele R, Banta J, Clark RT, Hayes SR, Edwards J, Cho TW, Wittlake WA (2007). Implementation of a bundle of quality indicators for the early management of severe sepsis and septic shock is associated with decreased mortality. Crit Care Med.

[11] Ferrer R, Artigas A, Levy MM, Blanco J, González-Díaz G, Garnacho-Montero J, Ibáñez J, Palencia E, Quintana M, de la Torre-Prados MV, Edusepsis Study Group (2008). Improvement in process of care and outcome after a multicenter severe sepsis educational program in Spain. J Am Med Assoc.

[12] Levy MM, Evans LE, Rhodes A (2018). The surviving sepsis campaign bundle: 2018 update. Crit Care Med.

[13] Seymour CW, Gesten F, Prescott HC, Friedrich ME, Iwashyna TJ, Phillips GS, Lemeshow S, Osborn T, Terry KM, Levy MM (2017). Time to treatment and mortality during mandated emergency care for Sepsis. N Engl J Med.

[14] Barbash IJ, Davis B, Kahn JM (2019). National performance on the medicare SEP-1 Sepsis quality measure. Crit Care Med.

[15] Kissoon N (2014). Sepsis guideline implementation: benefits, pitfalls and possible solutions. Crit Care.

[16] Kumar A, Roberts D, Wood KE, Light B, Parrillo JE, Sharma S, Suppes R, Feinstein D, Zanotti S, Taiberg L, Gurka D, Kumar A, Cheang M (2006). Duration of hypotension before initiation of effective antimicrobial therapy is the critical determinant of survival in human Septic shock. Crit Care Med.

[17] Arabi YM, Al-Dorzi HM, Alamry A, Hijazi R, Alsolamy S, Al Salamah M, Tamim HM, Al-Qahtani S, Al-Dawood A, Marini AM, Al-Ehnidi FH, Mundekkadan S, Matroud A, Mohamed MS, Taher S (2017). The impact of a multifaceted intervention including sepsis electronic alert system and sepsis response team on the outcomes of patients with sepsis and septic shock. Ann Intensive Care.

[18] Manaktala S, Claypool SR (2017). Evaluating the impact of a computerized surveillance algorithm and decision support system on sepsis mortality. J Am Med Inform Assoc.

[19] Nelson JL, Smith BL, Jared JD, Younger JG (2011). Prospective trial of real-time electronic surveillance to expedite early care of severe sepsis. Ann Emerg Med.

[20] Parshuram CS, Dryden-Palmer K, Farrell C, Gottesman R, Gray M, Hutchison JS, Helfaer M, Hunt EA, Joffe AR, Lacroix J, Moga MA, Nadkarni V, Ninis N, Parkin PC, Wensley D, Willan AR, Tomlinson GA, Canadian Critical Care Trials Groupthe EPOCH Investigators (2018). Effect of a pediatric early warning system on all-cause mortality in hospitalized pediatric patients: the EPOCH randomized clinical trial. J Am Med Assoc.

[21] Sawyer AM, Deal EN, Labelle AJ, Witt C, Thiel SW, Heard K, Reichley RM, Micek ST, Kollef MH (2011). Implementation of a real-time computerized sepsis alert in nonintensive care unit patients. Crit Care Med.

[22] Umscheid CA, Betesh J, van Zandbergen C, Hanish A, Tait G, Mikkelsen ME, French B, Fuchs BD (2015). Development, implementation, and impact of an automated early warning and response system for sepsis. J Hosp Med.

[23] Bedoya AD, Clement ME, Phelan M, Steorts RC, O'Brien C, Goldstein BA (2019). Minimal impact of implemented early warning score and best practice alert for patient deterioration. Crit Care Med.

[24] Harrison AM, Gajic O, Pickering BW, Herasevich V (2016). Development and implementation of sepsis alert systems. Clin Chest Med.

[25] Downing NL, Rolnick J, Poole SF, Hall E, Wessels AJ, Heidenreich P, Shieh L (2019). Electronic health record-based clinical decision support alert for severe sepsis: a randomised evaluation. BMJ Qual Saf.

[26] de Vries A, Draaisma JM, Fuijkschot J (2017). Clinician perceptions of an early warning system on patient safety. Hosp Pediatr.

[27] Ginestra JC, Giannini HM, Schweickert WD, Meadows L, Lynch MJ, Pavan K, Chivers CJ, Draugelis M, Donnelly PJ, Fuchs BD, Umscheid CA (2019). Clinician perception of a machine learning-based early warning system designed to predict severe sepsis and septic shock. Crit Care Med.

[28] Guidi JL, Clark K, Upton MT, Faust H, Umscheid CA, Lane-Fall MB, Mikkelsen ME, Schweickert WD, Vanzandbergen CA, Betesh J, Tait G, Hanish A, Smith K, Feeley D, Fuchs BD (2015). Clinician perception of the effectiveness of an automated early warning and response system for sepsis in an academic medical center. Ann Am Thorac Soc.

[29] Elish MC (2019). The Stakes of Uncertainty: Developing and Integrating Machine Learning in Clinical Care. Ethnographic Praxis in Industry Conference Proceedings.

[30] Sendak M, Elish M, Gao M, Futoma J, Ratliff W, Nichols M, Bedoya A, Balu S, O'Brien C (2020). The Human Body is a Black Box: Supporting Clinical Decision-making With Deep Learning. Proceedings of the 2020 Conference on Fairness, Accountability, and Transparency.

[31] Lin A, Sendak M, Bedoya A, Clement M, Brajer N, Futoma J, Bosworth H, Heller K, O'Brien C Evaluating sepsis definitions for clinical decision support against a definition for epidemiological disease surveillance. bioRxiv.

[32] Sendak MP, Ratliff W, Sarro D, Alderton E, Futoma J, Gao M, Nichols M, Revoir M, Yashar F, Miller C, Kester K, Sandhu S, Corey K, Brajer N, Tan C, Lin A, Brown T, Engelbosch S, Anstrom K, Elish MC, Heller K, Donohoe R, Theiling J, Poon E, Balu S, Bedoya A, O'Brien C (2020). Real-world integration of a sepsis deep learning technology into routine clinical care: implementation study. JMIR Med Inform.

[33] Futoma J, Hariharan S, Heller K Learning to detect sepsis with a multitask Gaussian process RNN classifier. arXivstat.

[34] Futoma J, Hariharan S, Sendak M, Brajer N, Clement M, Bedoya A, O?Brien C, Heller K (2017). An Improved Multi-Output Gaussian Process RNN with Real-Time Validation for Early Sepsis Detection. arXivstat.

[35] Endsley MR (2016). Toward a theory of situation awareness in dynamic systems. Hum Factors.

[36] Saunders B, Sim J, Kingstone T, Baker S, Waterfield J, Bartlam B, Burroughs H, Jinks C (2018). Saturation in qualitative research: exploring its conceptualization and operationalization. Qual Quant.

[37] Charmaz K (2006). Constructing Grounded Theory.

[38] Creswell J, Creswell J (2018). Research design: Qualitative, Quantitative, and Mixed Methods Approaches.

[39] Holden RJ, Karsh B (2010). The technology acceptance model: its past and its future in health care. J Biomed Inform.

[40] Asan O, Bayrak AE, Choudhury A (2020). Artificial intelligence and human trust in healthcare: focus on clinicians. J Med Internet Res.

[41] Kolachalama VB, Garg PS (2018). Machine learning and medical education. NPJ Digit Med.

[42] Sarwar S, Dent A, Faust K, Richer M, Djuric U, Van Ommeren R, Diamandis P (2019). Physician perspectives on integration of artificial intelligence into diagnostic pathology. NPJ Digit Med.

[43] Sendak MP, Gao M, Brajer N, Balu S (2020). Presenting machine learning model information to clinical end users with model facts labels. NPJ Digit Med.

[44] Bedoya A, Futoma J, Clement M, Corey K, Brajer N, Lin A, Simons M, Gao M, Nichols M, Balu S, Heller K, Sendak M, O'Brien C (2020). Machine learning for early detection of sepsis: an internal and temporal validation study. JAMIA Open.

[45] Lin H, Li R, Liu Z, Chen J, Yang Y, Chen H, Lin Z, Lai W, Long E, Wu X, Lin D, Zhu Y, Chen C, Wu D, Yu T, Cao Q, Li X, Li J, Li W, Wang J, Yang M, Hu H, Zhang L, Yu Y, Chen X, Hu J, Zhu K, Jiang S, Huang Y, Tan G, Huang J, Lin X, Zhang X, Luo L, Liu Y, Liu X, Cheng B, Zheng D, Wu M, Chen W, Liu Y (2019). Diagnostic efficacy and therapeutic decision-making capacity of an artificial intelligence platform for childhood cataracts in eye clinics: a multicentre randomized controlled trial. EClinicalMedicine.

[46] Keel S, Lee PY, Scheetz J, Li Z, Kotowicz MA, MacIsaac RJ, He M (2018). Feasibility and patient acceptability of a novel artificial intelligence-based screening model for diabetic retinopathy at endocrinology outpatient services: a pilot study. Sci Rep.

[47] Venkatesh, Morris, Davis, Davis (2003). User acceptance of information technology: toward a unified view. MIS Q.

[48] van de Velde S, Heselmans A, Delvaux N, Brandt L, Marco-Ruiz L, Spitaels D, Cloetens H, Kortteisto T, Roshanov P, Kunnamo I, Aertgeerts B, Vandvik PO, Flottorp S (2018). A systematic review of trials evaluating success factors of interventions with computerised clinical decision support. Implement Sci.

[49] Doraiswamy PM, Blease C, Bodner K (2020). Artificial intelligence and the future of psychiatry: insights from a global physician survey. Artif Intell Med.

[50] Blease C, Kaptchuk TJ, Bernstein MH, Mandl KD, Halamka JD, DesRoches CM (2019). Artificial intelligence and the future of primary care: exploratory qualitative study of UK general practitioners' views. J Med Internet Res.

[51] Laï MC, Brian M, Mamzer M (2020). Perceptions of artificial intelligence in healthcare: findings from a qualitative survey study among actors in France. J Transl Med.

